# Cytopathogenesis of Vesicular Stomatitis Virus Is Regulated by the PSAP Motif of M Protein in a Species-Dependent Manner

**DOI:** 10.3390/v4091605

**Published:** 2012-09-19

**Authors:** Takashi Irie, Yuliang Liu, Barbara S. Drolet, Elena Carnero, Adolfo García-Sastre, Ronald N. Harty

**Affiliations:** 1 Department of Virology, Institute of Biomedical and Health Sciences, Hiroshima University, 1-2-3 Kasumi, Minami-ku, Hiroshima 734-8551, Japan; Email: tirie@hiroshima-u.ac.jp; 2 Department of Pathobiology, School of Veterinary Medicine, University of Pennsylvania, 3800 Spruce St., Philadelphia, PA 19104, USA; Email: yuliang@vet.upenn.edu; 3 Arthropod-Borne Animal Diseases Research Unit, United States Department of Agriculture, Agricultural Research Service, 1515 College Ave., Manhattan, KS 66502, USA; Email: barbara.drolet@ars.usda.gov; 4 Department of Hepatology and Gene Therapy, University of Navarra, E-31009 Pamplona, Spain; Email: ecarnero@unav.es; 5 Department of Microbiology, Mount Sinai School of Medicine, 1 Gustave L. Levy Place, New York, NY 10029, USA; Email: adolfo.garcia-sastre@mssm.edu; 6 Department of Medicine, Division of Infectious Diseases, Mount Sinai School of Medicine, 1 Gustave L. Levy Place, New York, NY 10029, USA; 7 Global Health and Emerging Pathogens Institute, Mount Sinai School of Medicine, 1 Gustave L. Levy Place, New York, NY 10029, USA

**Keywords:** vesicular stomatitis virus, VSV recombinant, M protein, apoptosis, cytopathic, CPE, insect, persistence, motif

## Abstract

Vesicular stomatitis virus (VSV) is an important vector-borne pathogen of bovine and equine species, causing a reportable vesicular disease. The matrix (M) protein of VSV is multifunctional and plays a key role in cytopathogenesis, apoptosis, host protein shut-off, and virion assembly/budding. Our previous findings indicated that mutations of residues flanking the _37_PSAP_40_ motif within the M protein resulted in VSV recombinants having attenuated phenotypes in mice. In this report, we characterize the phenotype of VSV recombinant PS > A4 (which harbors four alanines (AAAA) in place of the PSAP motif without disruption of flanking residues) in both mice, and in *Aedes albopictus* C6/36 mosquito and *Culicoides sonorensis* KC cell lines. The PS > A4 recombinant displayed an attenuated phenotype in infected mice as judged by weight loss, mortality, and viral titers measured from lung and brain samples of infected animals. However, unexpectedly, the PS > A4 recombinant displayed a robust cytopathic phenotype in insect C6/36 cells compared to that observed with control viruses. Notably, titers of recombinant PS > A4 were approximately 10-fold greater than those of control viruses in infected C6/36 cells and in KC cells from *Culicoides sonorensis*, a known VSV vector species. In addition, recombinant PS > A4 induced a 25-fold increase in the level of C3 caspase activity in infected C6/36 cells. These findings indicate that the PSAP motif plays a direct role in regulating cytopathogenicity in a species-dependent manner, and suggest that the intact PSAP motif may be important for maintaining persistence of VSV in an insect host.

## 1. Introduction

Vesicular stomatitis virus (VSV) is an arbovirus and member of the *Rhabdoviridae* family from the *Mononegavirales* order of enveloped negative-strand RNA viruses. There are five genes encoded within the VSV genome in the order of 3′-N-P-M-G-L-5′. The matrix (M) protein is the smallest and most abundant structural protein that possesses multiple functions in virus assembly/budding, apoptosis, host protein shut-off, and pathogenicity [1,2,3,4,5,6,7,8,9,10,11,12,13,14,15,16,17,18,19,20,21,22,23,24,25]. A number of sequences and/or motifs have been identified in the M protein that contribute to the various functions mediated by M during virus infection [9,11,12,13,17,23,24,26,27,28,29,30,31,32,33,34,35]. For example, the highly conserved late (L) budding domain _24_PPPY_27_ present in the M protein mediates an interaction with host E3 ubiquitin ligase Nedd4 via its WW-domains to facilitate virus egress [9,11,13,19,26,27,31]. In addition to the PPPY motif, a second motif of interest is the downstream _37_PSAP_40_ motif. The PSAP domain has been implicated in playing a role in both budding and pathogenesis of VSV [12,24,27,31]. Indeed, the PSAP motif was found recently to mediate an interaction with host Tsg101 and contribute to virion egress in a cell type dependent manner [31]. We had reported previously that mutations in sequences immediately flanking the PSAP motif resulted in VSV recombinants that were attenuated *in vitro* and *in vivo* [12,24,27]. Thus, the PSAP region appears to be critical for multiple functions of VSV M during replication in mammalian cells.

In this report, we sought to determine the phenotype of a virus carrying a direct mutation of the PSAP motif in a small animal model of infection. In addition, since VSV is an arbovirus that must replicate in both an insect and mammalian host, we sought to determine whether the PSAP motif of M is important for replication/pathogenesis in insect cell lines. Interestingly, we found that disruption of the PSAP motif resulted in an attenuated phenotype in mice; however, disruption of the PSAP motif resulted in the opposite phenotype in the *Aedes albopictus* C6/36 mosquito cell line and the *Culicoides sonorensis* KC cell line. Indeed, the PSAP mutant virus exhibited enhanced CPE and viral titers in insect cells compared to those of control viruses. Our findings suggest that the mechanism responsible for enhanced CPE and higher virus titers observed in insect cells may be due to an enhanced apoptotic effect, since the PSAP mutant virus was shown to induce a 25-fold higher level of caspase C3 activity in the C6/36 mosquito cell line compared to that induced by control viruses. These findings suggest that the PSAP motif is an important regulator of cytopathogenicity induced by VSV, and that disruption of the PSAP motif leads to either an attenuated or cytopathic phenotype that is species dependent.

## 2. Results

### 2.1. VSV Recombinant PS > A4 Is Attenuated in Mice

**Figure 1:**
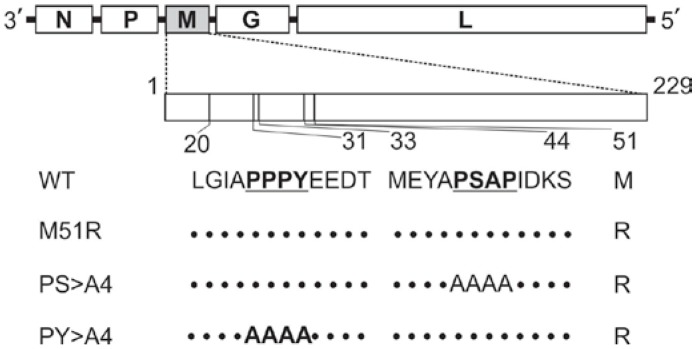
Diagram of the Vesicular stomatitis virus (VSV) genome with the M gene (highlighted in gray) expanded. The amino acid positions and sequences of the PPPY (amino acids 20 to 31) and PSAP (amino acids 33 to 44) regions of M are shown for the indicated viruses. The PPPY and PSAP motifs are shown in bold and underlined. The dotted lines indicate that the VSV‑wild type (VSV‑WT) M sequence is maintained.

VSV recombinant PS > A4 was generated using reverse genetics [12,27] to contain four alanines in place of the PSAP motif within the M protein (Figure 1). For comparison, the corresponding amino acid sequence from VSV-wild type (VSV-WT), VSV host shutoff mutant (M51R), and VSV-PY > A4 (budding defective mutant containing four alanines in place of the PPPY motif within the M protein) are also depicted (Figure 1). M51R was used as a control in most experiments since the PS > A4 mutation was built into the M51R background (Figure 1). Our previous findings revealed that recombinant PS > A4 produced plaque size measurements and titers in BHK-21 cells that were virtually identical to those produced by both VSV-WT and M51R viruses [12,27]. In contrast, recombinant PY > A4 produced plaques with reduced diameters and titers that were on average 10-fold lower than those produced by control viruses [12,27].

**Figure 2:**
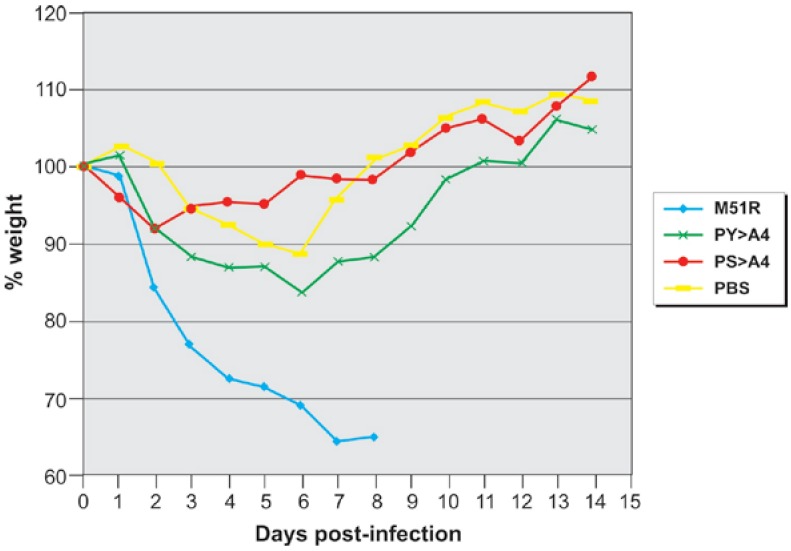
Graph of weight change in virus infected mice. Graphic depiction of the average % weight loss/gain for mock-infected mice (PBS, yellow), or mice infected with M51R (blue), PY > A4 (green), or PS > A4 (red). Weights were determined post-infection on a daily basis for a two-week period.

We first sought to determine whether recombinant PS > A4 displayed an attenuated phenotype in a mouse model of VSV infection. Toward this end, groups of eight 6-week old BALB/c mice were infected with recombinant PS > A4 or with control viruses (VSV-WT or M51R) and then assessed for weight loss, survival, and virus replication in both the lungs and brain. Mice were inoculated with 10^7^ p.f.u. of the indicated virus and body weight was recorded on a daily basis for a period of 14 days (Figure 2). All mice infected with the parental M51R virus exhibited consistent and significant weight loss from day 0 to day 8, whereas mice that were mock-infected (PBS) or infected with either PS > A4 or PY > A4 lost <20% of their body weight and ultimately gained body weight during later days post‑infection (Figure 2).

**Figure 3:**
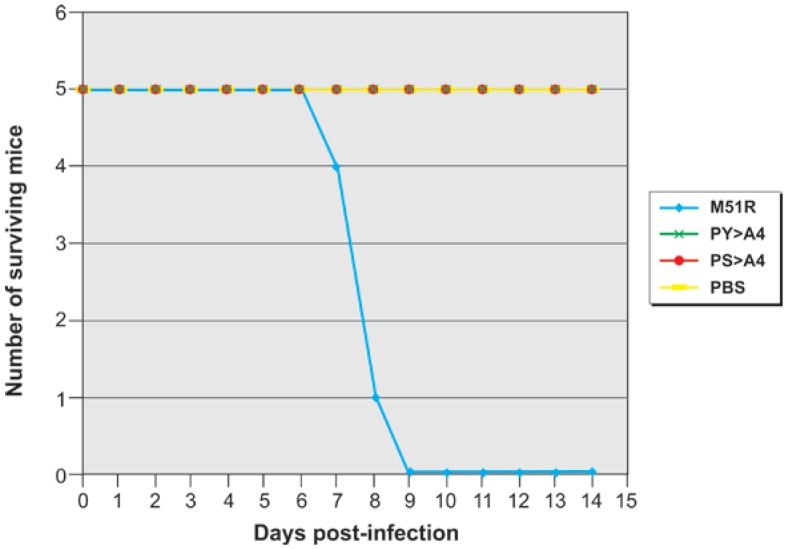
Survival of virus infected mice. Groups of eight mice were mock infected (PBS, yellow), or infected intranasally with 10^7^ p.f.u. of M51R (blue), PY > A4 (green), or PS > A4 (red), and survival was monitored on a daily basis for a two-week period. All data points for PBS, PY > A4, and PS > A4 fall on the same horizontal line.

All mice infected with the parental M51R virus eventually succumbed to the infection by day 9 post-infection (Figure 3). In contrast, all mice that were mock-infected or infected with VSV recombinants PS > A4 or PY > A4 survived during the 14 day period (Figure 3). 

**Figure 4:**
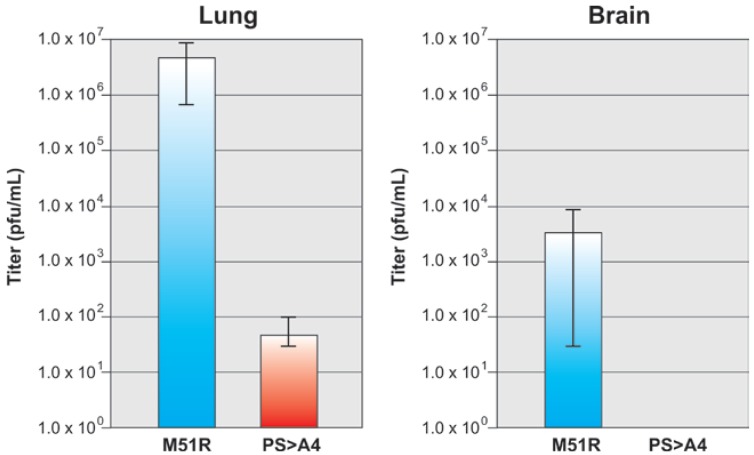
Replication of virus in the lungs and brains of infected mice. Three mice initially infected with 10^7^ p.f.u. of M51R or PS > A4 were sacrificed at 2 d.p.i., and the average viral titers (p.f.u./mL) from lung and brain homogenates were determined using a standard plaque assay on BHK-21 cells performed in triplicate.

Three mice from each group were euthanized on day 2 for virus titration of lung and brain tissues using a standard plaque assay on BHK-21 cells (Figure 4). The mean viral titer of M51R in the lungs was 7.4 × 10^6^ p.f.u./mL whereas that of recombinant PS > A4 was approximately 7.0 × 10^1^ p.f.u./mL (Figure 4). In brain samples taken from infected mice, M51R replication was detected in all three mice at an average titer of 3.2 × 10^3^ p.f.u./mL (Figure 4). Interestingly, replication of recombinant PS > A4 was undetectable in the brains from all three sacrificed mice (Figure 4). It should be noted that replication of recombinant PY > A4 was detected in the lungs of all three sacrificed mice at an average titer of 1.2 × 10^5^ p.f.u./mL (data not shown), and was detected in the brain of only 1 of 3 sacrificed animals (data not shown). The pathogenic nature of M51R may be related to the initial inoculum, age and strain of the mice, and/or a bolus of virus in the lung. These data indicate that mutagenesis of the PSAP motif to AAAA leads to a phenotype of severe attenuation compared to that of the parental virus in this small animal model of infection.

### 2.2. VSV Recombinant PS > A4 Is Pathogenic in Insect Cells

**Figure 5:**
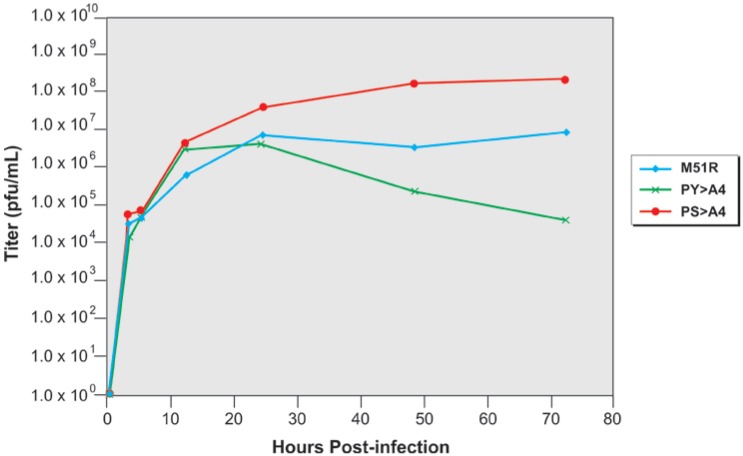
Growth curves in mosquito C6/36 cells. Mosquito C6/36 cells were infected with M51R (blue), PY > A4 (green), or PS > A4 (red) at an MOI of 50, and virus released into the supernatants at the times indicated was harvested and quantified by standard plaque assay on BHK-21 cells. A representative growth curve is shown.

Since VSV is an arbovirus that must replicate in both an insect and mammalian host, we sought to determine whether recombinant PS > A4 also exhibited an attenuated phenotype in insect cells as compared to control viruses. Toward this end, mosquito C6/36 cells were infected with M51R, PY > A4, or PS > A4 at an MOI of 50, and virus released into the supernatant was collected at various times post-infection and quantified by standard plaque assay on BHK-21 cells performed in triplicate. To our surprise, we found that recombinant PS > A4 reproducibly replicated to titers in C6/36 cells that were up to 1.0 log greater than that of M51R, and up to 3.0 logs greater than that of recombinant PY > A4 (Figure 5). Titers for all three viruses steadily increased with time up to approximately 24 h.p.i., at which time titers of PS > A4 continued to rise to approximately 1.0 × 10^8^ p.f.u./mL at 70 h post-infection, whereas titers of M51R leveled off at approximately 1.0 × 10^7^ p.f.u./mL and titers of PY > A4 began to decline to approximately 1.0 × 10^5^ p.f.u./mL at 70 h post-infection (Figure 5).

**Figure 6:**
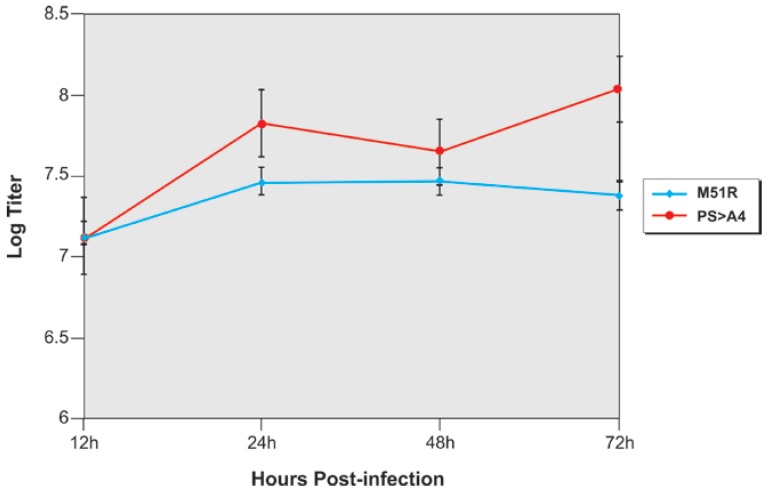
Growth curves in *Culicoides sonorensis* cells. *Culicoides* cells were infected with M51R (blue) and PS > A4 (red) at an MOI of 3.0, and virus released into the supernatants at the times indicated was harvested and quantified by standard plaque assay on Vero cells performed in triplicate.

In addition to using mosquito C6/36 cells, we also infected the KC cell line from *Culicoides sonorensis*, a known VSV vector species, with M51R and recombinant PS > A4 at an MOI of 3.0 (Figure 6). Consistent with the results obtained using C6/36 cells, recombinant PS > A4 replicated to titers approximately 10-fold higher in KC cells compared to those observed for M51R over a 72 h time period (Figure 6). Together, these data indicate that titers of recombinant PS > A4 were consistently higher than those of the parental M51R virus in insect cell lines.

**Figure 7:**
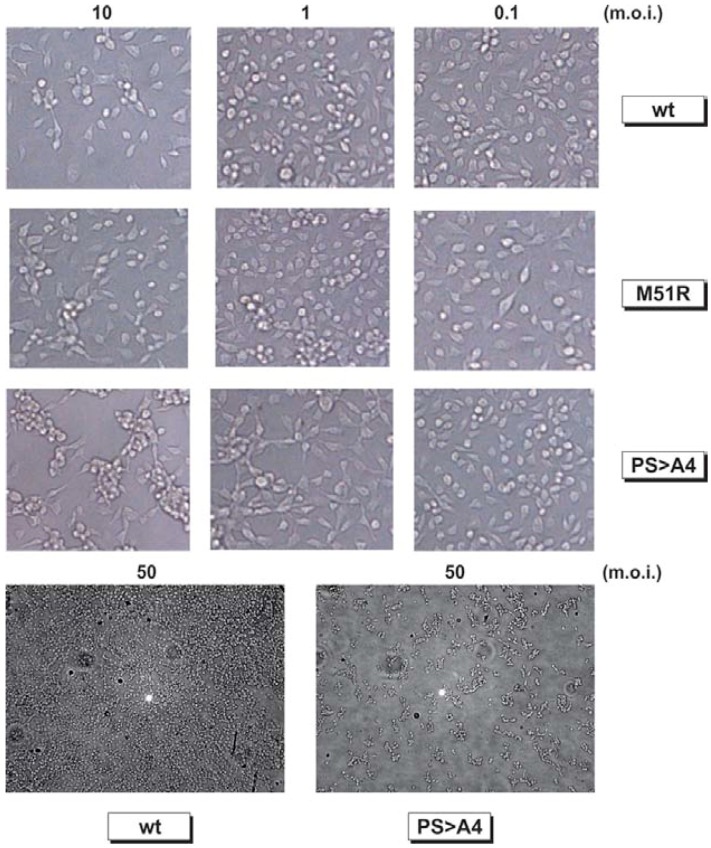
Microscopic analysis of virus-induced CPE in mosquito C6/36 cells. Mosquito C6/36 cells were infected with either VSV-WT, M51R, or PS > A4 at an MOI of 0.1, 1.0, or 10, and CPE was observed by light microscopy (200× magnification) at 24 h p.i. In a separate experiment, C6/36 cells were infected with either VSV-WT or PS > A4 at an MOI of 50, and CPE was observed by light microscopy (50× magnification) at 24 h post‑infection.

Next, we used light microscopy to observe virus-induced CPE at 24 h.p.i. in monolayers of mosquito C6/36 cells infected with VSV-WT, M51R, or PS > A4 at MOIs of either 0.1, 1.0, 10, or 50 (Figure 7). There was no appreciable difference between CPE induced by VSV-WT or M51R viruses in C3/36 cells at all MOIs tested (Figure 7). In contrast, robust CPE was observed in cells infected with recombinant PS > A4 at the lower MOIs tested, and even more evident at MOIs of 10 and 50 (Figure 7). CPE induced by recombinant PS > A4 was dramatically distinct from that observed for VSV-WT or M51R viruses (Figure 7). Indeed, mosquito C6/36 cells infected with recombinant PS > A4 virus showed severe morphological alterations; including cell rounding, aggregation and detachment (Figure 7). Taken together, the titer and CPE data indicate that disruption of the PSAP motif of M results in a virus having enhanced growth properties and robust cytopathology in C6/36 and KC cells compared to those of parental control viruses: a phenotype completely opposite to that observed in a mammalian host.

### 2.3. Virus-Induced Caspase 3 Activity in Mosquito C6/36 Cells

**Figure 8:**
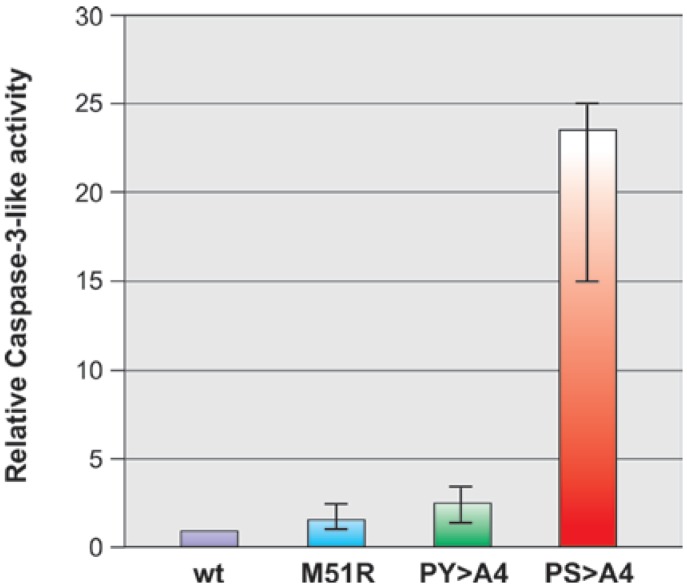
Activated caspase-3 levels in virus infected C6/36 cells. Mosquito C6/36 cells were infected with VSV-WT, M51R, PY > A4, or PS > A4 at an MOI of 50 for 24 h. At 24 h post-infection, cells were harvested and assayed for caspase-3-like activity. Each value represents the average of three independent experiments. The level of activated caspase-3 observed for VSV-WT was normalized to 1.0.

To begin to address the mechanism by which recombinant PS > A4 establishes enhanced replication and cytopathology in insect cells, we sought to determine whether this cytopathic phenotype associated with recombinant PS > A4 may be linked to virus-induced apoptosis in mosquito C6/36 cells. While it is well established that VSV M plays a central role in the induction of apoptosis in mammalian cells in a caspase-3 dependent manner [4,5,6,20,21,36], a comparable role for M in insect cells is less well defined. Activated levels of caspase-3 were measured by colorimetric assay in mosquito C6/36 cells that were mock-infected, or infected with either VSV-WT, M51R, PY > A4, or PS > A4 at an MOI of 50 at 24 h p.i. (Figure 8). The level of activated caspase-3 induced by VSV-WT was normalized to 1.0 (Figure 8). As expected, the average level of activated caspase-3 induced by infection with M51R was virtually identical to that induced by VSV-WT (Figure 8). Similarly, the average level of activated caspase-3 induced by recombinant PY > A4 was within 2-fold of that observed for the parental M51R virus (Figure 8). In stark contrast to these results, the average level of activated caspase-3 induced by infection with recombinant PS > A4 was approximately 25-fold greater than that induced by VSV-WT and approximately 12.5-fold greater than that induced by M51R (Figure 8). These findings indicate that disruption of the PSAP motif dramatically affected the levels of activated caspsae-3 induced by VSV infection of mosquito C6/36 cells, and suggest that the enhanced cytopathology associated with recombinant PS > A4 may be linked to enhanced stimulation of the insect apoptotic pathway.

## 3. Discussion

M protein of VSV is the major structural protein of the virus and plays a key role in assembly/budding, host protein shutoff, and cytopathology in mammalian cells [1,2,3,4,5,6,7,8,9,10,11,12,13,14,15,16,17,18,19,20,21,22,23,24,25]. The functional contributions of the conserved PSAP motif and flanking residues of VSV M to virus replication and pathogenesis in mammalian cells have been the focus of several recent investigations [12,24,27,31]. For example, Obiang *et al.* reported recently that the PSAP motif can recruit host Tsg101 and in doing so contributes to the efficient egress of VSV in a cell-type dependent manner [31]. In addition, our findings suggest that the PSAP motif is an important species-dependent regulator of cytopathology.

Although the growth properties of the PS > A4 recombinant have been characterized previously in cell culture [12,27], data presented in this report are the first to characterize this recombinant’s pathogenic potential in a mammalian host and in insect cells. The PS > A4 recombinant exhibited a highly attenuated phenotype in mice compared to those observed for VSV-WT and M51R control viruses in terms of weight loss, survival, and viral titers in the lungs and brains of infected animals. The attenuated phenotype observed for the PS > A4 recombinant in mice is likely not due simply to a defect in virus budding since the overall phenotypic profiles for PS > A4 and the budding defective PY > A4 were distinct in both cell culture [12,27] and in mice (this report). Moreover, VSV recombinants having mutations in sequences immediately flanking the PSAP motif have been shown to be budding competent [12,27], yet were attenuated in mice compared to control viruses [24].

Virtually nothing is known about the contribution of the PSAP motif to VSV replication, cytopathology, and apoptosis in insect cells. One of the most intriguing findings of this study was the highly pathogenic nature of the PS > A4 recombinant in C6/36 mosquito cells compared to control viruses. Indeed, titers of the PS > A4 recombinant measured in C6/36 cells at later time points were approximately 1 log greater than that of parental M51R, and approximately 3 logs greater than that of PY > A4. Similarly, titers of the PS > A4 recombinant were consistently higher than those produced by M51R in the *Culicoides sonorensis* KC cell line. The finding that titers of PY > A4 were 10–100 fold lower than those of M51R at later times post-infection suggest that the PPPY motif may possess a bonafide L-domain function in insect cells as it does in mammalian cells. In correlation with the observed higher titers, the PS > A4 recombinant also produced extensive and robust CPE in C6/36 cells infected at various MOIs compared to that observed for VSV-WT, M51R, and PY > A4. Taken together, these findings indicate that disruption of the PSAP motif of VSV M results in a virus having enhanced cytopathic properties, which suggests that the intact PSAP motif may be critical for VSV to maintain persistence or a non-cytopathic phenotype in the insect vector.

Although additional studies are required to fully elucidate the mechanism by which PS > A4 induces CPE in insect cells, we did observe a significant increase in the levels of activated caspase-3 in mosquito cells infected with PS > A4 compared to those observed in mosquito cells infected with control viruses. In contrast, the levels of activated capase-3 were identical in Hela cells infected with either M51R or PS > A4 at an MOI of 10 (Irie and Harty, data not shown). It is possible that enhanced apoptotic activity and cell death may contribute to enhanced CPE and titers of recombinant PS > A4 in C6/36 cells [37]. Again, it is tempting to speculate that an intact PSAP motif may be important for regulating or dampening the apoptotic pathway in insect cells, thereby reducing cell death and establishing a state of persistence. Indeed, a link among VSV persistence, M protein mutations, and levels of caspase-3 in human neural cells has been reported [38]. Further characterization of the molecular mechanism by which the PSAP motif regulates cytopathology and apoptosis in insect cells is warranted.

## 4. Experimental Section

### 4.1. Cell and Viruses

*Aedes albopictus* mosquito C6/36 cells were propagated in Leibovitz’s medium (Invitrogen; Carlsbad, CA, USA) containing 10% heat-inactivated fetal bovine serum (FBS, Invitrogen) and penicllin/streptomycin (Invitrogen) and incubated at 28 °C under 5% CO_2_. *Culicoides sonorensis* KC cells were propagated in Schneider’s Drosophila medium (Gibco, Grand Island, NY) containing 10% FBS at 30 °C. Wild-type (WT), M51R, PS > A4 and PY > A4 viruses have been described previously [12,13,24,28]. All VSV recombinants were propagated in BHK-21 cells and titrated by standard plaque assay on BHK-21 [27] or Vero cells [39].

### 4.2. Analysis of Cytopathic Effect and Growth Kinetics of Recombinant Viruses

Mosquito C6/36 cells in six-well plates were infected with the indicated viruses at MOIs of 0.1, 1.0 10 or 50. After incubation for 1 h at 28 °C, the medium was removed, and cells were washed with 1× phosphate-buffered saline (PBS) twice and incubated with fresh Leibovitz’s media containing 5% FBS at 28 °C. At 24 h p.i., cells were examined for CPE by light microscopy. For growth curves on C6/36 or KC cell lines, virions released into the supernatant at the indicated time points were quantified by titration using a standard plaque assay on BHK-21 cells [27].

### 4.3. Detection of Activated Caspase-3 Activity

Mosquito C3/36 cell in six-well plates were mock-infected (negative control) or infected with the indicated virus at an MOI of 50. After incubation for 1 h, the culture medium was removed, and cells were washed with 1 × PBS twice and then inoculated with fresh medium containing 5% FBS. At 24 h post-infection, approximately 5 × 10^6^ cells were harvested by centrifugation and washed two times with 1 × PBS. Cells were suspended in 50 μL of chilled Cell Lysis buffer (BioVision) and assayed using a Caspase-3 Colorimetric Assay kit (BioVision) according to the manufacturer’s instructions.

### 4.4. Pathogenicity in Mice

Groups of eight 6-week old female BALB/c mice were mock-infected with PBS, or infected intranasally with 10^7^ p.f.u./mL of VSV-WT, M51R, PY > A4, or PS > A4. Body weight measurements were recorded on a daily basis over a two-week period, and survival was monitored and plotted over a two-week period. At 2 days post-infection, three mice were sacrificed, and viral titers were determined from lung and brain homogenates of sacrificed animals using a standard plaque assay on BHK-21 cells.

## 5. Conclusions

We conclude that mutagenesis of the PSAP motif of VSV M to four alanines results in a virus that possesses a highly attenuated phenotype in mice compared to control viruses. These findings correlate well with previous studies showing that mutations in sequences immediately flanking the PSAP motif also result in viruses having an attenuated phenotype in mice [24]. We also conclude from these and prior studies [12,24,27] that the overall phenotypic profile of recombinant PS > A4 is distinct from that of the budding defective recombinant PY > A4, suggesting that the role of the PSAP motif in regulating cytopathology, and perhaps budding [31], is species dependent. Lastly, we conclude that recombinant PS > A4 displays a highly cytopathic profile in mosquito C6/36 cells, in contrast to its highly attenuated phenotype in a mammalian host. Thus, the PSAP motif appears to be a key regulator of cytopathogenesis of VSV in different host species, and may be crucial for regulating a lytic *versus* persistent outcome of infection.
